# Optimising electrogenerated chemiluminescence of quantum dots via co-reactant selection

**DOI:** 10.1007/s00216-016-9557-1

**Published:** 2016-04-25

**Authors:** Rebekah Russell, Alasdair J. Stewart, Lynn Dennany

**Affiliations:** Department of Pure and Applied Chemistry, Technology and Innovation Centre, University of Strathclyde, 99 George Street, Glasgow, G1 1RD UK

**Keywords:** Electroanalytical methods, Electrochemiluminescence, Quantum dots

## Abstract

We demonstrate that for quantum dot (QD) based electrochemiluminescence (ECL), the commonly used co-reactant does not perform as effectively as potassium persulfate. By exploiting this small change in co-reactant, ECL intensity can be enhanced dramatically in a cathodic-based ECL system. However, TPA remains the preferential co-reactant-based system for anodic ECL. This phenomenon can be rationalised through the relative energy-level profiles of the QD to the co-reactant in conjunction with the applied potential range. This work highlights the importance of understanding the co-reactant pathway for optimising the application of ECL to bioanalytical analysis, in particular for near-infrared (NIR) QDs which can be utilised for analysis in blood.

**Graphical Abstract:**
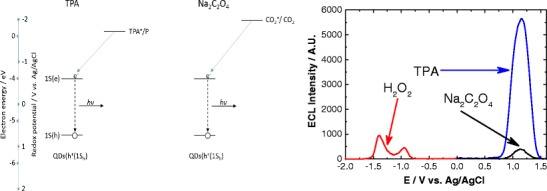
Optimising ECL Production Through Careful Selection of Co-Reactions Based on Energetics Involved

Optimising ECL Production Through Careful Selection of Co-Reactions Based on Energetics Involved

## Introduction

The application of electrochemiluminescence (ECL) in research and commercial applications has been predominantly focused on ruthenium complexes that displayed intense, stable signals in both organic and aqueous media [1–4]. The vast majority of these systems are based upon the classic [Ru(bpy)_3_]^2+^-tri-*n*-propylamine (TPA) co-reactant system, and the development of new luminophores and alternative co-reactants have attracted much attention. Following the discovery of ECL emission from silicon quantum dots (QDs) [5], the focus of investigations shifted towards nanomaterials that displayed size-tunable emission and enhanced optical and electronic properties [6]. The vast majority of these works focused on materials that emitted in the visible region, resulting in a good understanding of the ECL behaviour of these materials.

The ECL of visible region QDs has been studied extensively, which has been shown to produce an ECL response with a variety of co-reactants [7–10]. This has allowed the development of a number of ECL biosensors that use visible region QDs as labels [11–14]. Near-infrared (NIR) QDs are of increasing interest owing to their emission wavelength that lies outside the absorption range of biological fluids and tissue. The potential benefits of NIR-emitting species in biosensing and imaging applications have been well documented because of their improved penetrability through biological samples and reduced tissue autofluorescence [15, 16]. This can provide more detailed and better-defined images for deep tissue imaging. For biosensing, it opens up opportunities for development of systems with detection directly from whole blood samples, negating the requirement for time-consuming and expensive sample preparation procedures.

Currently, no such investigations into the behaviour of NIR QDs in different systems have been carried out, with the majority of work focused on cathodic NIR ECL with potassium persulfate co-reactant [17–19]. Only a single example exists of anodic NIR ECL [20–22], and there are currently no documented ECL systems that utilise NIR-emitting QDs and no additional co-reactant (termed co-reactant-free systems). Therefore, the ECL characteristics of these QDs have not been determined in a variety of systems, which has prevented a full understanding of their ECL behaviour. Investigation into these properties should supplement the electrochemical characterisation of these QDs and could aid in the development of a greater variety of NIR ECL biosensors.

NIR-emitting QDs are beginning to emerge as leaders in this field as a result of their excellent optical properties, large surface-to-volume ratio and surface modification opportunities [23]. They have successfully been used within in vivo imaging studies [24–28]; however, there has been limited work on their application within ECL biosensing platforms [19, 20]. This has recently been shown for the determination of dopamine in whole blood, highlighting the significance of NIR QDs for biosensing [21]. This research demonstrates the flexibility of NIR QDs, which can generate an ECL signal with a variety of co-reactant systems. Therefore, the development was the optimisation of these conditions to obtain the most sensitive, responsive and stable ECL signal. This has not been done previously with NIR QDs, and thus there is a clear requirement for such investigations.

The aims of this work were to investigate the ECL characteristics of NIR QDs in a variety of co-reactant systems and determine the likely mechanisms of their response and to determine the optimal co-reactant for a defined application. Although this work is specific to NIR QD ECL, the insights found can be applied to any QD ECL-based system.

## Experimental

### Apparatus

Electrochemical measurements were carried out using a CH instrument model 760D electrochemical analyser. All experiments were carried out using a conventional three-electrode assembly, consisting of a 3 mm diameter GC working electrode (unless otherwise stated), Pt wire counter electrode and Ag/AgCl reference electrode. Working electrodes were cleaned by successive polishing using 1, 0.3 and 0.05 μM alumina slurry, followed by sonication in ethanol and water, respectively, for 30 min. The electrodes were then dried under a flow of N_2_ gas. Cyclic voltammetry (CV) was carried out at a scan rate of 100 mV s^**−**1^ and sample interval of 1 mV across a potential range outlined in each figure. Measurements involving simultaneous detection of light and current utilised a CH instrument model 760D connected to a Hamamatsu H10723-20 PMT. The input voltage to the PMT was +5 V, and the control voltage was set between 0.5 and 1.05 V depending on the required sensitivity. The scan rate was 100 mV s^**−**1^ (unless otherwise stated). During electrochemical experiments, the cell was kept in a light-tight Faraday cage in a specially designed holder configuration where the working electrode was positioned directly above the PMT window. All measurements were made at room temperature.

### Materials

Core-shell CdSeTe/ZnS QDs (Qdot® 800 ITK™ organic quantum dots, 1 μM in decane) were purchased from Invitrogen. 2-(Dimethylamino)ethanthiol (DAET), Nafion® 117 solution, chitosan (medium molecular weight, 75–85 % deacetylated), phosphate-buffered saline (PBS, pH 7.4), potassium persulfate (K_2_S_2_O_8_), hydrogen peroxide (H_2_O_2_), tripropylamine (TPA), sodium oxalate (Na_2_C_2_O_4_), tris acetate–EDTA (TAE) buffer, 4-morpholineethanesulfonic acid hydrate (MES), sodium bicarbonate, sulfuric acid (H_2_SO_4_) and sodium hydroxide (NaOH) were all purchased from Sigma-Aldrich and used as received. All other reagents used were of analytical grade, and all solutions were prepared in Milli-Q water (18 mΩ cm).

### Methods

#### Preparation of water-soluble CdSeTe/ZnS core-shell QDs

The method followed was similar to that developed by Woelfle and Claus [29, 30]. 0.5 mL of 0.5 M DAET in methanol was mixed with 0.25 mL of the CdSeTe/ZnS QDs in decane (1 μM). N_2_ was bubbled through the solution for 5 min, which was then sealed and left stirring overnight in the dark at room temperature. The QDs were then precipitated with an excess of acetone followed by centrifugation at 5000 rpm for 6 min. The filtrate was removed and the precipitate was re-dispersed in 0.25 mL of distilled water. These water-soluble QDs were centrifuged for a further 6 min at 3000 rpm to remove any impurities and then stored in darkness at 4 °C.

#### Preparation of CdSeTe/ZnS core-shell QD–polymer composite films

A 0.1 % stock solution of chitosan in 1 % acetic acid was prepared. The QD/chitosan composite was prepared by mixing aliquots of the water-soluble QDs with the chitosan solution in a 1:1 (*v*/*v*) ratio. Three microliters of this composite was then carefully cast onto the electroactive portion of a GC electrode and allowed to dry for 1 h at 4 °C. A film of bare QDs and QD–Nafion was prepared in the same manner, with water and 0.1 % Nafion 117 in MeOH/H_2_O (4/1) used instead of chitosan, respectively. The polymer concentration was altered by changing the concentration of its stock solution pre-dilution with the QDs. QD concentration in the film was altered by mixing the water-soluble QDs with a suitable volume of water prior to mixing in a 1:1 (*v*/*v*) ratio with chitosan.

#### Preparation of co-reactant solutions

Co-reactant solutions of TPA, Na_2_C_2_O_4_, H_2_O_2_ and K_2_S_2_O_8_ were prepared in 0.1 M PBS (pH 7.4) at the concentrations outlined in each figure.

## Results and discussion

### Estimation of HOMO and LUMO energy levels

**Fig. 1:**
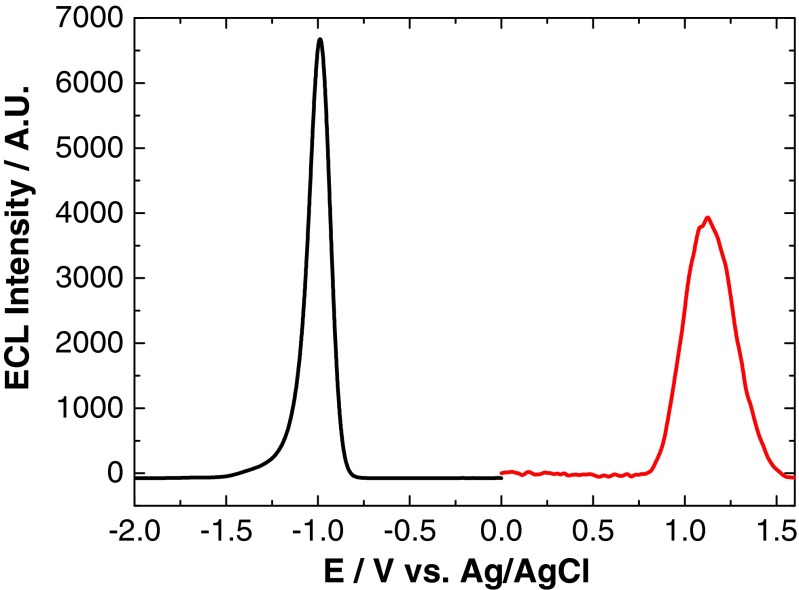
ECL response of an 800 nm QD/chitosan film in 1 mM TPA (*red*) and 1 mM K_2_S_2_O_8_ (*black*) at a scan rate of 100 mV s^−1^ over the potential range –2 ≤ *ν* ≤ 2 V vs. Ag/AgCl

The onset of QD oxidation and reduction has previously been used to estimate the highest occupied molecular orbital (HOMO)–lowest unoccupied molecular orbital (LUMO) gap [25, 31], also known as the quasi-particle gap. Often, the quasi-particle gap estimated in this way can be unreliable, as the true oxidation and reduction potentials of the QDs cannot always be detected. Therefore, it was proposed that the onset potential for ECL could be used as a more accurate estimate of these potentials, as the rate-determining step for ECL generation is the oxidation or reduction of QDs. Figure 1 shows the anodic and cathodic ECL profiles of NIR QDs. The oxidative and reductive ECL onset potentials for the QDs and the HOMO–LUMO energy gap are shown in Table 1.

**Table 1:** Reduction and oxidation ECL onset potentials and resulting HOMO–LUMO energy gap for a series of QDs in the presence of a co-reactant

QD/nm	Reduction ECL onset/V vs. Ag/AgCl	Oxidation ECL onset/V vs. Ag/AgCl	HOMO–LUMO energy gap/eV
800	−0.75	0.75	1.50

**Fig. 2:**
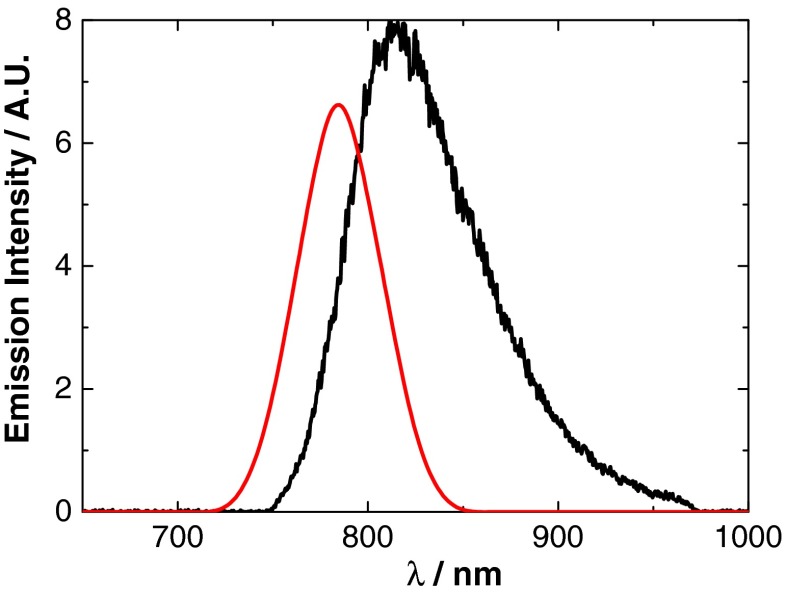
Emission profiles of 800 nm QDs from optically induced (*red*) and ECL (*black*) processes

**Fig. 3:**
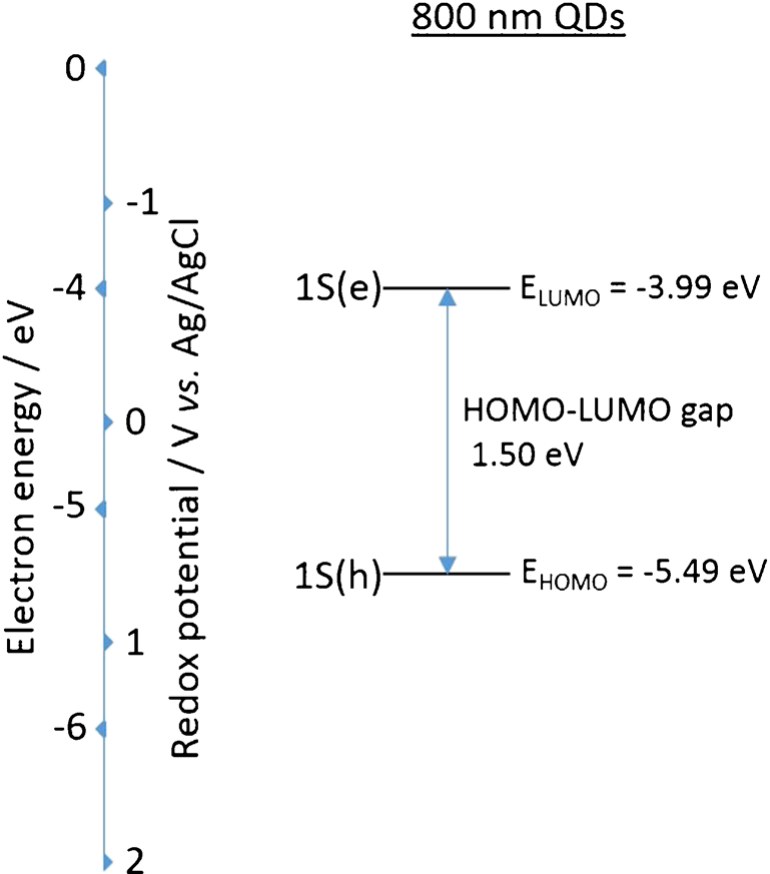
Energy level diagram for 800 nm CdSeTe/ZnS QDs based on their reductive and oxidative ECL onset potentials and HOMO–LUMO energy gap

The estimated HOMO–LUMO energy gap of 800 nm QDs (1.50 eV) is in good agreement with the optical *E*_*g*_ of 1.569 eV from optically induced emission and 1.529 eV from ECL emission (see Fig. 2). This confirms that ECL emission is originating from the QD core. The proposed electronic structure of these NIR QDs is outlined in Fig. 3. The HOMO and LUMO energy levels are calculated from the reduction and oxidation ECL onset potentials (the energy level of Ag/AgCl in a vacuum is calculated as −4.74 eV) [32, 33].

HOMO energy is in excellent agreement with that obtained from differential pulse voltammetry (DPV), whilst LUMO energy is 0.70 eV less energetic when using ECL onset potentials. This data suggests electron injection into the 1S(e) quantum confined orbital of the NIR QDs is taking place at a more positive potential than that observed using voltammetric techniques. The similarity between optical *E*_*g*_ and the HOMO–LUMO energy gap calculated from ECL onset potentials suggests this method of electronic structure estimation is more accurate than that using CV or DPV.

### Co-reactant assessment

In order to develop a highly sensitive ECL system, a number of co-reactants were examined to ensure maximum performance for these NIR QDs. As biomedical diagnostics continually drive towards improved biosensor sensitivities, this is a key parameter in the development of any sensing system.

**Fig. 4:**
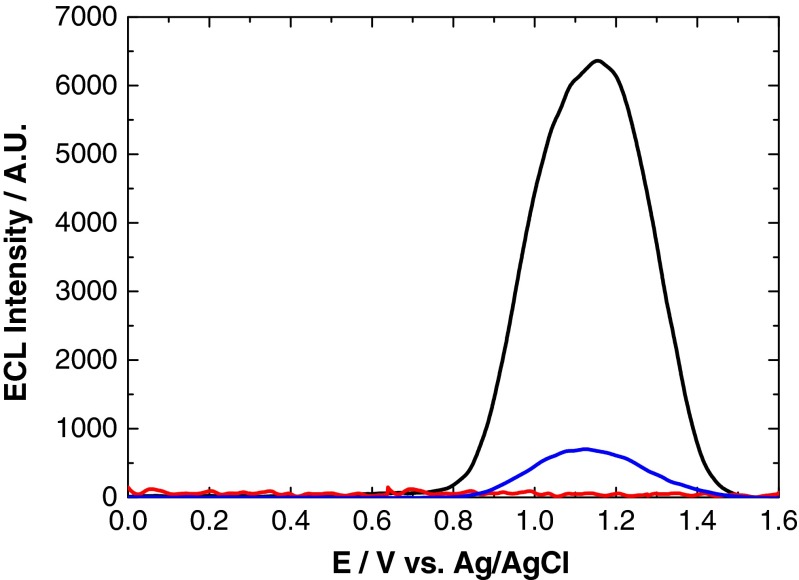
ECL response of 800 nm QD/chitosan film in 0.1 M PBS (*red*) + 1 mM Na_2_C_2_O_4_ (*blue*) and + 1 mM TPA (*black*) at a scan rate of 100 mV s^−1^ over the potential range 0.5 ≤ *ν* ≤ 1.6 V vs. Ag/AgCl

Anodic ECL involves an oxidative–reductive system in which a hole is injected into the 1S(h) energy level of the QD through heterogeneous electron transfer with the electrode. This is followed by electron injection into the 1S(e) energy level of this charged particle via homogeneous electron transfer with a co-reactant that has sufficient reducing power. Tripropylamine (TPA) and sodium oxalate (Na_2_C_2_O_4_) are typical anodic ECL co-reactants that have been studied extensively within ruthenium-containing systems [2, 3, 34–37]. However, generation of an ECL signal between these co-reactants and NIR QDs has not yet been investigated. Figure 4 shows the ECL profile of NIR QDs with TPA and Na_2_C_2_O_4_ co-reactants, as well as in a solution of 0.1 M PBS (co-reactant-free system).

**Fig. 5:**
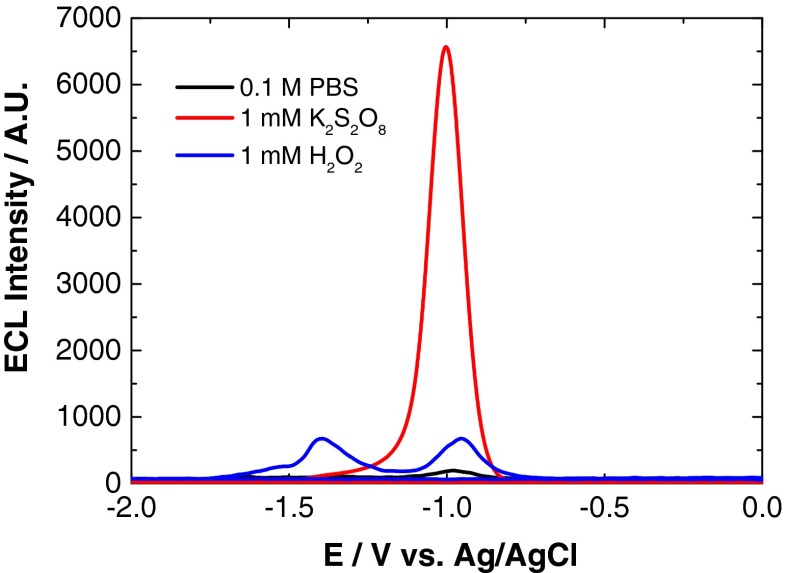
ECL response of 800 nm QD/chitosan film in 0.1 M PBS (*black*), 1 mM H_2_O_2_ (*blue*) and 1 mM K_2_S_2_O_8_ (*red*) at a scan rate of 100 mV s^−1^ over the potential range −2 ≤ *ν* ≤ 0 V vs. Ag/AgCl

Cathodic ECL involves formation of ECL precursor species through reduction at the electrode surface, followed by homogenous electron transfer between these species to generate an excited state (reductive–oxidative system). For QDs, an electron is injected into the 1S(e) energy level of their conduction band at a potential governed by their size. For emission of an ECL signal, hole injection into the 1S(h) orbital of this charged QD is then required, which is achieved through interaction with a strong oxidising agent created via reduction and decomposition of a suitable co-reactant species. Typical co-reactant species capable of forming such reactive intermediates include hydrogen peroxide (H_2_O_2_) and potassium persulfate (K_2_S_2_O_8_) [1, 18, 38–43]. Figure 5 shows the QD ECL profile with H_2_O_2_ and K_2_S_2_O_8_ co-reactants, and in PBS (co-reactant-free system).

Cathodic ECL was observed with K_2_S_2_O_8_ and H_2_O_2_ co-reactants and with the co-reactant-free system. Both H_2_O_2_ and 0.1 M PBS exhibit a double peak profile with the onset of reductive ECL peak 1 at −0.75 V (ECL-1) and the onset of peak 2 at −1.15 V (ECL-2). Maximum intensity of these peaks is reached at −1.00 and −1.35 V, respectively. The strongest ECL signal was obtained with K_2_S_2_O_8_, which displayed a single reductive ECL peak with the onset at −0.75 V and peak maximum at −1.00 V.

As mentioned, in the presence of H_2_O_2_, two ECL peaks were present, which has been observed previously [21]. The initial peak, ECL-1, was shown to result from the interaction of QDs with radical oxygen species (ROS) created following O_2_ reduction at the electrode surface. ECL-2 is produced following the one-electron reduction of H_2_O_2_ to produce OH^·^, which can then interact with QDs to generate ECL as outlined in Eqs. 1–7. Previous investigations have shown that ECL-2 is more sensitive to the dissolved H_2_O_2_ and thus should be chosen to detect H_2_O_2_ for the production of ECL through the following electrochemical reactions [21]:

1 $$ \mathrm{Q}\mathrm{D}\mathrm{s} + 1{\mathrm{e}}^{-}\to \mathrm{Q}\mathrm{D}\mathrm{s}\left({\mathrm{e}}^{-}{1}_{\mathrm{Se}}\right) $$

2 $$ {\mathrm{H}}_2{\mathrm{O}}_2 + 1{\mathrm{e}}^{-}\to\ {\mathrm{O}\mathrm{H}}^{-} + {\mathrm{O}\mathrm{H}}^{\cdot } $$

3 $$ \mathrm{Q}\mathrm{D}\mathrm{s}\left({\mathrm{e}}^{-}{1}_{\mathrm{Se}}\right) + {\mathrm{H}}_2{\mathrm{O}}_2\to\ \mathrm{Q}\mathrm{D}\mathrm{s} + {\mathrm{O}\mathrm{H}}^{-} + {\mathrm{O}\mathrm{H}}^{\cdot } $$

4 $$ {\mathrm{OH}}^{\cdot } + \mathrm{Q}\mathrm{D}\mathrm{s}\to {\mathrm{OH}}^{-} + \mathrm{Q}\mathrm{D}\mathrm{s}\left({\mathrm{h}}^{+}{1}_{\mathrm{Sh}}\right) $$

5 $$ \mathrm{Q}\mathrm{D}\mathrm{s}\left({\mathrm{e}}^{-}{1}_{\mathrm{Se}}\right) + {\mathrm{OH}}^{\cdot}\to {\mathrm{OH}}^{-} + \mathrm{Q}\mathrm{D}\mathrm{s}* $$

6 $$ \mathrm{Q}\mathrm{D}\mathrm{s}\left({\mathrm{e}}^{-}{1}_{\mathrm{Se}}\right) + \mathrm{Q}\mathrm{D}\mathrm{s}\left({\mathrm{h}}^{+}{1}_{\mathrm{Sh}}\right)\to \mathrm{Q}\mathrm{D}\mathrm{s}* $$

7 $$ \mathrm{Q}\mathrm{D}\mathrm{s}*\to \mathrm{Q}\mathrm{D}\mathrm{s} + \mathrm{h}\upnu \left(800\ \mathrm{nm}\right) $$

**Fig. 6:**
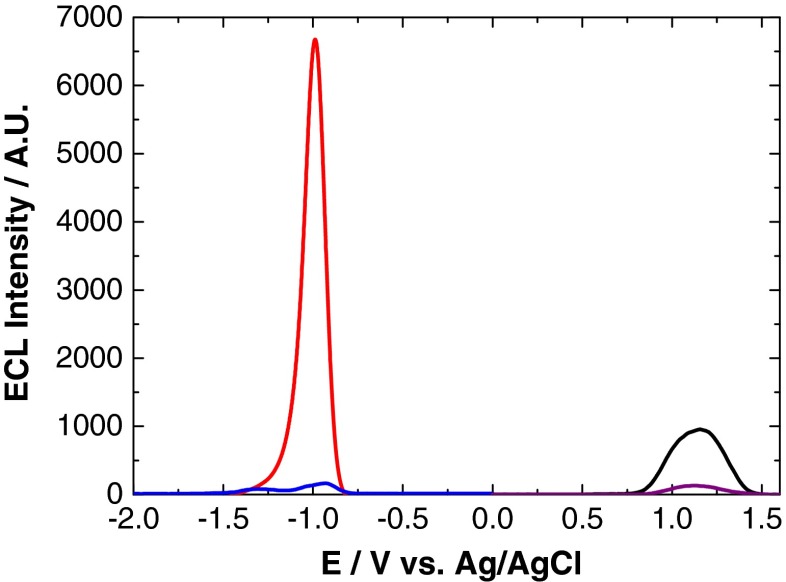
ECL response of 800 nm QD/chitosan film with 1 mM K_2_S_2_O_8_ (*red*), 1 mM H_2_O_2_ (*blue*), 1 mM TPA (*black*) and 1 mM Na_2_C_2_O_4_ (*purple*) at a scan rate of 100 mV s^−1^ over the potential range −2 ≤ *ν* ≤ 1.6 V vs. Ag/AgCl

This shows that a NIR QD ECL response can be generated in the presence of commonly used cathodic (K_2_S_2_O_8_ and H_2_O_2_) and anodic (TPA and Na_2_C_2_O_4_) region co-reactants, which were shown to enhance ECL intensities compared to co-reactant-free systems. Therefore, these co-reactants were selected for investigation with the aim of determining which system provided optimal ECL performance. A comparison of the ECL response from these co-reactants is shown in Fig. 6.

**Fig. 7:**
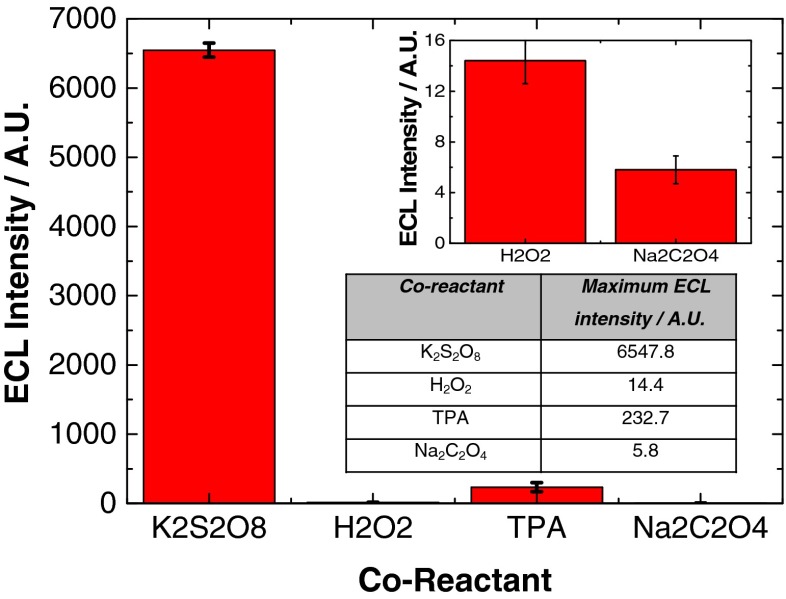
Maximum ECL intensity of 800 nm QD/chitosan film in a selection of co-reactant systems. The *inset* shows the lower response of H_2_O_2_ and Na_2_C_2_O_4_ for clarity with the averaged results also shown

It is clearly evident from Fig. 6 that K_2_S_2_O_8_ generates the most intense ECL response from NIR QDs that have been confined to the electrode surface. This is followed by TPA, H_2_O_2_ and Na_2_C_2_O_4_. Figure 7 shows the maximum ECL intensity attained with each co-reactant.

**Fig. 8:**
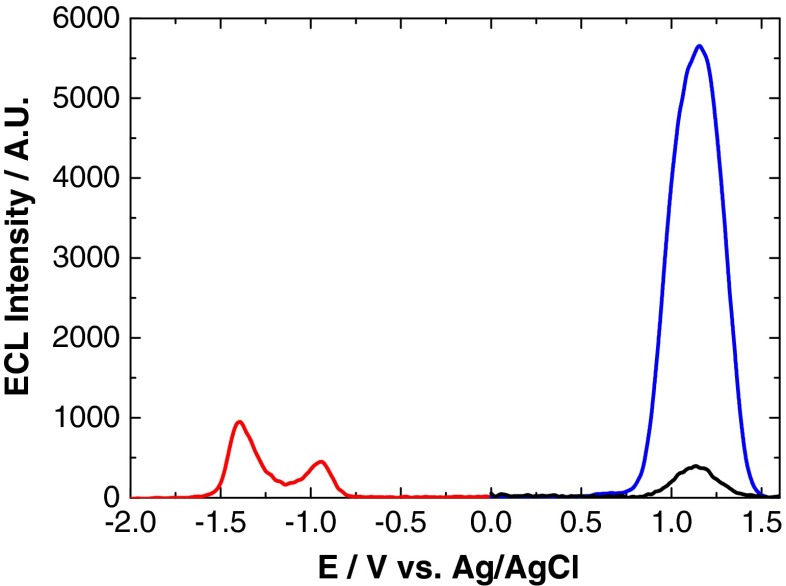
ECL response of 800 nm QD/chitosan film with 1 mM H_2_O_2_ (*red*), 1 mM TPA (*blue*) and 1 mM Na_2_C_2_O_4_ (*black*) at a scan rate of 100 mV s^**−**1^ over the potential range −2 ≤ *ν* ≤ 1.6 V vs. Ag/AgCl

This data illustrates that maximum ECL intensity was obtained with K_2_S_2_O_8_, which was over 450 times greater than with the alternative cathodic co-reactant, H_2_O_2_. It was 30 times greater than with TPA and over 1100 times greater than with Na_2_C_2_O_4_. With anodic co-reactants, maximum ECL intensity was 40 times greater in TPA compared to Na_2_C_2_O_4_. Figure 8 shows a clearer image of the ECL response with H_2_O_2_, TPA and Na_2_C_2_O_4_ using more sensitive PMT settings, confirming the trend in sensitivity is TPA > H_2_O_2_ > Na_2_C_2_O_4_.

**Fig. 9:**
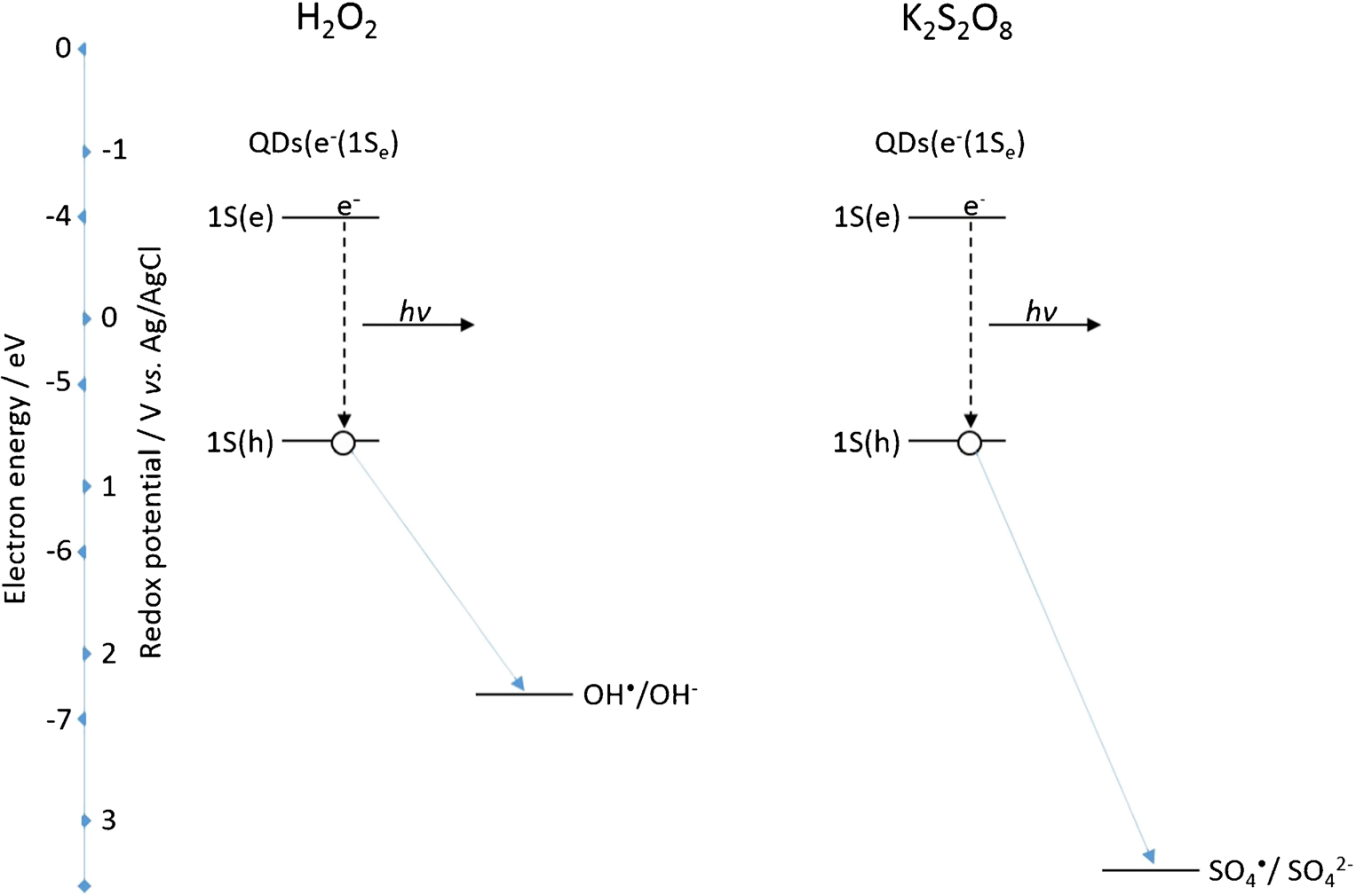
Significant energy-level interactions and resulting ECL process of 800 nm QDs with H_2_O_2_ and K_2_S_2_O_8_ co-reactants

As mentioned, cathodic ECL required the formation of excited state QDs [33]; this occurs through the interaction with a suitably strong reducing agent—SO_4_^·**−**^ and OH^·^—for K_2_S_2_O_8_ and H_2_O_2_ co-reactants, respectively. Rapid band-edge recombination of this excited state QD dominates over any oxidation processes, protecting destruction of the QDs following hole injection and allowing efficient ECL production [44]. The rate of this intermolecular electron transfer between a negatively charged QD and the oxidising agent is a major factor in the generated ECL intensity [45]. Therefore, the strength of the oxidising agent has a critical impact on the observed ECL intensity. The standard redox potential (vs. Ag/AgCl) for the SO_4_^·**−**^/SO_4_^2**−**^ couple is approximately 3.16 V [46], whereas for the OH^·^/OH^**−**^ couple, it is 2.16 V (vs. Ag/AgCl) at physiological pH [47]. Figure 9 shows a comparison of the energetics of these species with the QD HOMO and LUMO levels and their interactions during the ECL process.

Both oxidising species are capable of hole injection into the 1S(h) quantum confined orbital of the NIR QDs. This can be seen both in Fig. 9 as well as the fact that an ECL response is observed with both co-reactants. The greater oxidising strength of SO_4_^·**−**^ compared to OH^·^ results in a more rapid rate of QD hole injection and therefore a more rapid rate of excited state QD formation. This manifests itself as an increase in ECL intensity with the K_2_S_2_O_8_ system. It must be noted that the double peak nature of the ECL profile in H_2_O_2_ will likely influence the ECL intensity of the H_2_O_2_-sensitive peak. This is because the concentration of QDs (e^**−**^(1S_e_)) for interaction with OH^·^ will have been diminished following consumption during generation of peak 1.

**Fig. 10:**
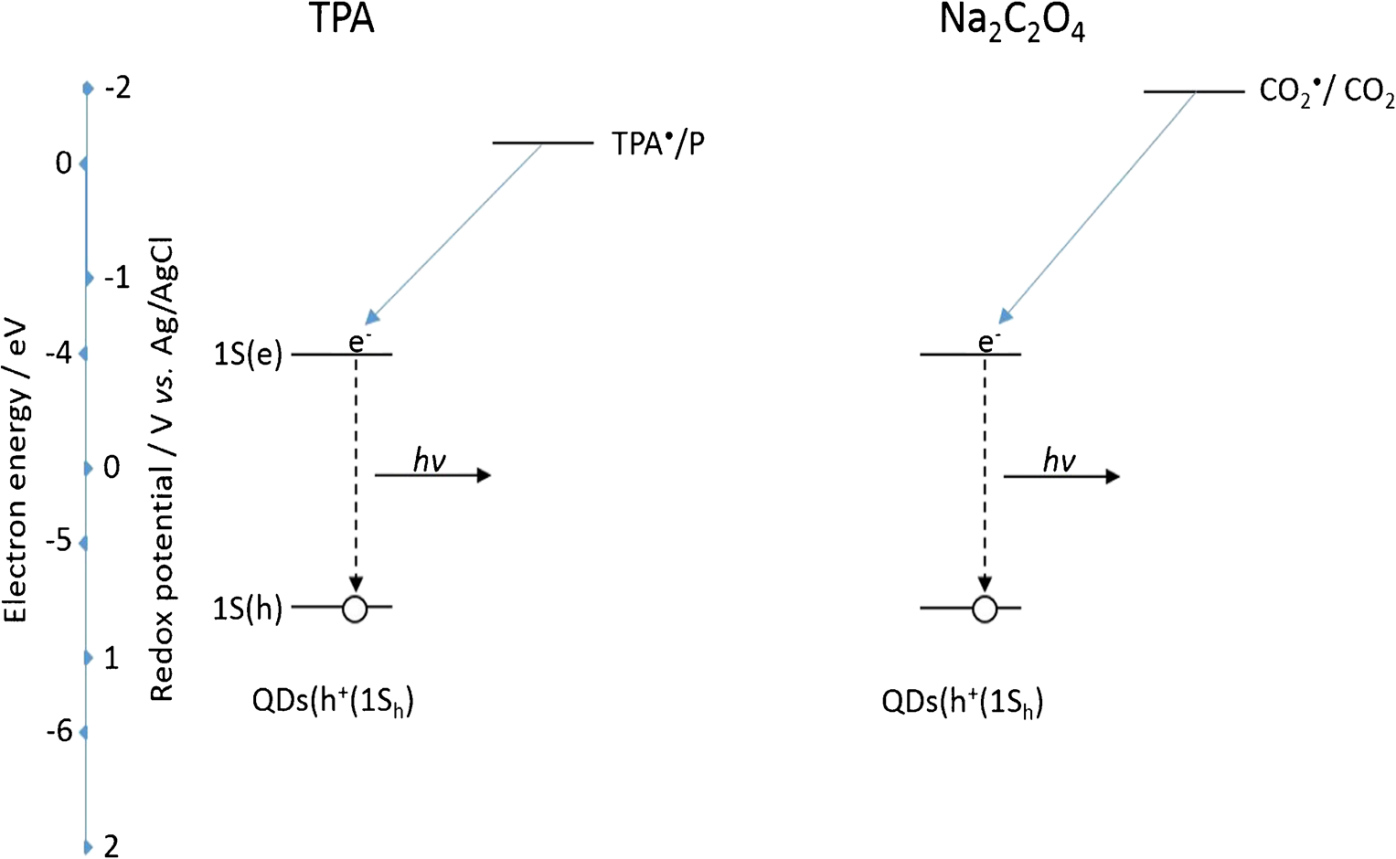
Significant energy-level interactions and resulting ECL process of 800 nm QDs with TPA and Na_2_C_2_O_4_ co-reactants

For anodic ECL, one factor affecting intensity is the ability of the electrogenerated co-reactant species to inject an electron into the 1S(e) energy level of oxidised QDs. Figure 10 shows a comparison of the energetics of these co-reactant species (TPA^·^ and CO_2_^·**−**^) with the QD HOMO and LUMO levels and their interactions during the ECL process. The standard redox potential of TPA^·^/P, where P is the product of TPA^·^ oxidation, is approximately −1.70 V (vs. Ag/AgCl) [2] and that of CO_2_^·**−**^/CO_2_ is approximately −2.00 V (vs. Ag/AgCl) [48].

The stronger reducing power of CO_2_^·**−**^ compared to TPA^·^ does not result in a more intense ECL signal (Fig. 7), as would be expected due to faster homogenous electron transfer with QDs (h^+^(1S_h_)). This means another factor is affecting excited state formation in this system. This is related to consumption of QDs (h^+^(1S_h_)) during electrogeneration of CO_2_^·**−**^. The result is that ECL intensity of the QD/TPA system is significantly greater, as electrogeneration of TPA^·^ can occur directly at the electrode surface, even though homogeneous electron transfer kinetics in this system are likely slower.

These results clearly show that maximum NIR QD ECL sensitivity is achieved in the cathodic region with K_2_S_2_O_8_ co-reactant. Development of NIR QD ECL systems that require maximum sensitivity should therefore focus on cathodic ECL with this co-reactant. The data have also shown that H_2_O_2_ and TPA are suitable co-reactants, however, a limited response with Na_2_C_2_O_4_ suggests it is unsuitable for use in this system.

## Conclusion

Significantly, this is the first detailed investigation into the optimal conditions for generation of ECL from NIR QDs based on co-reactant selection, which are likely to play a key role in future development of ECL biosensors [3]. In the future, this research will aid in the selection of suitable co-reactants for achieving optimal biosensor response from these NIR QDs. The main point of significance from this research is the far superior sensitivity of K_2_S_2_O_8_ co-reactant ECL compared to other common co-reactants, indicating that this should be used preferentially to obtain the most intense response. For any QD-based system, this requires consideration of the electrode platform, whether anodic or cathodic responses are required, the onset and energy-level interactions resulting from the QD and co-reactant which can be based upon the data presented here. It should be noted that the energy levels for the QDs are specific to their size and will impact on the selection of an appropriate co-reactant.

However, the detection of both cathodic and anodic ECL responses demonstrates the versatility of these NIR QDs, which should allow their use in a wide variety of sensing systems and to expand the application of ECL-based systems into biological samples such as blood and tissues. Overall, these investigations have outlined the best electrochemical system for generation of an intense NIR QD ECL response. This provides the framework for further NIR QD ECL biosensor development.
